# Analysis of polymorphisms, promoter methylation, and mRNA expression profile of maternal and placental P53 and P21 genes in preeclamptic and normotensive pregnant women

**DOI:** 10.1186/s12929-019-0586-x

**Published:** 2019-11-08

**Authors:** Mahdiyeh Harati-Sadegh, Leila Kohan, Batool Teimoori, Mehrnaz Mehrabani, Saeedeh Salimi

**Affiliations:** 1 Genetic of Non-Communicable Disease Research Center, Zahedan University of Medical Sciences, Zahedan, Iran; 2 Department of Biology, Arsanjan Branch, Islamic Azad University, Arsanjan, Iran; 3 Department of Obstetrics and Gynecology, School of Medicine, Zahedan University of Medical Sciences, Zahedan, Iran; 4 Physiology Research Center, Institute of Basic and Clinical Physiology Sciences, Kerman University of Medical Sciences, Kerman, Iran; 5 Department of Clinical Biochemistry, School of Medicine, Zahedan University of Medical Sciences, Zahedan, Iran; 6 Cellular and Molecular Research Center, Zahedan University of Medical Sciences, Zahedan, Iran

**Keywords:** P21, P53, Placenta, Polymorphism, Preeclampsia, Methylation

## Abstract

**Background:**

Preeclampsia (PE), as a multisystem disorder, is associated with maternal hypertension and proteinuria. Apoptosis seems to be involved in the pathophysiology of PE, although its precise pathogenic mechanisms are not well established. In this study, we aimed to identify the association between maternal *TP53*-rs1042522, *P21*-rs1801270, and *P21*-rs1059234 polymorphisms and PE. In addition, we examined the effects of promoter methylation and *TP53* and *P21* polymorphisms on placental mRNA expression in PE women.

**Methods:**

The blood of 226 PE women and 228 normotensive pregnant women was examined in this study. In addition, the placentas were genotyped in 109 PE and 112 control women. The methylation status was assessed by a methylation-specific PCR assay, while mRNA expression was examined via Quantitative Real Time PCR.

**Results:**

The maternal and placental *P21*-rs1801270 CA genotype had a significant association with the reduced risk of PE. In the dominant, recessive, and allelic models, maternal/placental *P21*-rs1059234 polymorphism had no statistically significant association with the risk of PE. On the other hand, the reduced risk of PE was associated with maternal, but not placental *TP53*-rs1042522 polymorphism in the dominant and recessive models. The maternal and placental *P21*-rs1801270 polymorphism was associated with PE risk. The maternal *P21* T_rs__1059234_C_rs__1801270_ haplotype was associated with 3.4-fold increase in PE risk, However the maternal *P21* T_rs__1059234_A_rs 1801270_ haplotype and placental C_rs1059234C_A _rs1801270_ haplotype led to 0.5 and 0.4-fold decrease in PE risk, respectively. PE women showed 5.6 times higher levels of placental mRNA expression of *TP53 gene*, although it was not associated with rs1042522 polymorphism. The relative placental mRNA expression of *P21* gene was 0.2 in PE women. It was also 2.4 times higher in individuals with rs1801270CA genotype than those with AA genotype. The hyper-methylation of *P21* and *TP53* genes in the promoter region was associated with a 3.4-fold and 3-fold increase in PE risk, respectively. However, no association was found between P21 and TP53 mRNA expression and promoter methylation.

**Conclusion:**

In conclusion, *P21*-rs1801270 and *TP53*-rs1042522 polymorphisms were involved in reduced risk of PE. *P21*-rs1801270 was associated with decreased P21 mRNA expression. The hyper-methylation of *P21* and *TP53* genes in the promoter region was associated with a higher PE risk.

## Background

Preeclampsia (PE) is a multisystem disorder, characterized by maternal hypertension and proteinuria. PE has been detected in 3–5% of all pregnancies around the world [1, 2]. This disorder may progress to eclampsia (convulsive form) due to late presentation, delayed diagnosis, and delayed treatment. It is therefore necessary to identify reliable hallmarks for early diagnosis of PE and develop efficient treatment strategies [3]. The precise pathogenic mechanisms underlying PE are still undetermined. Nevertheless, some important factors in PE development involve insufficient development of the placenta, immune maladaptation, oxidative stress, thrombosis, and placental ischemia. In addition, there are genetic factors in these components, which contribute to these pathogenic changes [4, 5]. In the trophoblast life cycle, normal proliferation and apoptosis are considered necessary. Aberrant cell turnover occurs in PE pregnancies, leading to increased apoptosis in placental trophoblasts [6]. The placental size and uteroplacental blood flow are limited due to abnormal cytotrophoblast cell differentiation during spiral artery invasion in uterine; therefore, the needs of the growing fetus are not met. In addition, placental hypoxia can be led to increased apoptosis in syncytiotrophoblast cells and necrosis [7]. The intracellular mechanisms of apoptosis in the PE placenta remain unknown [8]. *TP53* as a tumor suppressor contributes to the progression of cell cycle and apoptosis [9]. P53 protein, as a major transcription factor, is involved in the regulation of cell apoptosis, growth arrest, and DNA repair under cell stress conditions. To activate downstream target genes, p53 is phosphorylated and acetylated at different sites [10]. During complicated pregnancies, the level of p53 increases in the placenta, highlighting its role in the apoptosis of trophoblasts [11]. Downstream transcription of elements contributing to cell apoptosis and cycle arrest (e.g., p21) is promoted by increased level of p53 [8]. The *TP53* gene on chromosome 17 encodes a 53-kDa protein composed of 393 amino acids. Functional effects have been attributed to various single nucleotide polymorphisms (SNPs) of *TP53* gene. At codon 72, a G > C substitution characterizes the *TP53* gene polymorphism (P72R, rs1042522), which is found in the transactivation domain of the p53 protein with possible effects on the protein activity [12]. P21 (p21Cip1), as an important cell cycle regulator in G1 and S phases, is involved in the pathogenesis of various diseases [13]. Overall, rs1801270 (C98A), as the most common *P21* gene polymorphism, is a C > A substitution at codon 31 of the P21 protein. Accordingly, DNA binding of zinc finger motif and P21 expression may alter. In addition, rs1059234 (C70T) is another *P21* polymorphism, which is located downstream the stop codon in the 3′-untranslated region. This site is involved in cell proliferation, differentiation, and tumor suppression [14]. Several studies have indicated both genes and apoptotic pathways are important in the regulation on PE [15, 16]. However, no studies have examined the association of *P21* gene polymorphisms with PE development. In addition, the association between the *TP53* polymorphisms and PE has been less highlighted [17]. On the other hand, some studies have confirmed a relationship between methylation of various genes and PE [2, 18]. These results suggest that epigenetic modifications such as DNA methylation affect gene expression and can act as susceptibility factors in various diseases, including PE. DNA methylation as a complex process depends on environmental factors and genomic background [19]. Although the pathogenesis of PE is closely associated with the methylation status of the relevant genes [20], the association between placental promoter methylation of *P21*/*TP53* gene and PE risk remains unexamined. In the present study, we examined the association of maternal and placental *TP53* (rs1042522) and *P21* (rs1801270 and rs1059234) gene polymorphisms with the risk of PE development. Then, we examined the effects of placental *P21*-rs1801270, *P21*-rs1059234, and *TP53*-rs1042522 polymorphisms and placental DNA methylation of *P21* /*TP53* gene (promoter regions) on P21/TP53 mRNA expression.

## Materials and methods

### Subjects

In this study, a total of 228 healthy pregnant women and 226 PE women were recruited from Ali-ebn-Abi Taleb Hospital, affiliated to Zahedan University of Medical Sciences. PE was characterized by hypertension and proteinuria. Proteinuria was defined as ≥0.3 g/24 h or ≥ + 1 protein on a urine dipstick after the 20th gestational week, and hypertension was described as systolic blood pressure (SBP) ≥ 140 mmHg or diastolic blood pressure (DBP) ≥ 90 mmHg on two or more assessments (within at least six-hour intervals) [21]. Absence of proteinuria or hypertension and no increase in blood pressure were the study inclusion criteria for control group. On the other hand, autoimmune diseases, renal disorders, chronic hypertension, cancer, collagen vascular disorders, and thrombosis were among the study exclusion criteria. Following delivery, the placentas of 112 normotensive women and 109 PE women, as well as the blood samples of all study participants were collected.

### Sample preparation

After collecting peripheral blood samples (2 mL) from mothers, they were kept in a freezer at − 20 °C. Placental tissues were collected after childbirth. To remove fetal and maternal blood, phosphate-buffered saline was used for washing the tissue samples at 4 °C. The tissue samples were kept at − 80 °C for the extraction of DNA and RNA.

### Genetic analysis

The salting-out method was used for extracting genomic DNA from peripheral blood samples, treated with EDTA. A DNA extraction kit (DynaBio, Takapoozist, Iran) was also used to extract DNA from placental tissues according to manufacturer’s instruction. The PCR-RFLP assay was carried out for the detection of *TP53* (rs1042522) and *P21* (rs1801270 and rs1059234) polymorphisms, as described by Yaghmaei et al. [12] and Salimi et al. [14], respectively. The annealing temperature, primer sequences, and fragment size are presented in Table 1. To confirm genotyping quality, we carried out the randomly repeated genotyping for about 30% of the samples and the findings were in accordance to the preceding genotyping results.

**Table 1:** The primer sequences, annealing temperature and fragment sizes for molecular analysis

	Method	Primer Sequence 5′ ➔ 3’	PCR Product (bp)	Tm °C
P21-rs1059234	PCR-RFLP^a^	Forward: CCCAGGGAAGGGTGTCCTG Reverse: GGGCGGCCAGGGTATGTAC	298	64
P21-rs1801270	PCR-RFLP	Forward: GTCAGAACCGGCTGGGGATG Reverse: CTCCTCCCAACTCATCCCGG	272	64
TP53-rs1042522	PCR-RFLP	Forward: GTCCCAAGCAATGGATGAT Reverse: CAAAAGCCAAGGAATACACG	551	61
P21 promoter region	MSP^b^	FM: TACGCGAGGTTTCGGGATC RM: CCCTAATATACAACCGCCCCG	174	60
		FU: GGATTGGTTGGTTTGTTGGAATTT RU: ACAACCCTAATATACAACCACCCCA	164	
P53 promoter region	MSP	FM: GTAGTTTGAACGTTTTTATTTTGGC RM: CCTACTACGCCCTCTACAAACG	115	60
		FU: GTAGTTTGAATGTTTTTATTTTGGT RU: CCTACTACACCCTCTACAAACA	115	
P21	Real-time PCR	Forward: GCTCTGCTGCAGGGGACAGC Reverse: TCTGCCGCCGTTTTCGACCC	136	60
P53	Real-time PCR	Forward: GAGCTGAATGAGGCCTTGGA Reverse: CTGAGTCAGGCCCTTCTGTCTT	151	60
β-Actin	Real-time PCR	Forward: CCTGGCACCCAGCACAAT Reverse: GCCGATCCACACGGAGTACT	70	60

^a^ Polymerase chain reaction–restriction fragment length polymorphism ^b^ Methylation Specific PCR

### DNA bisulfite conversion and methylation-specific PCR

**Fig. 1:**
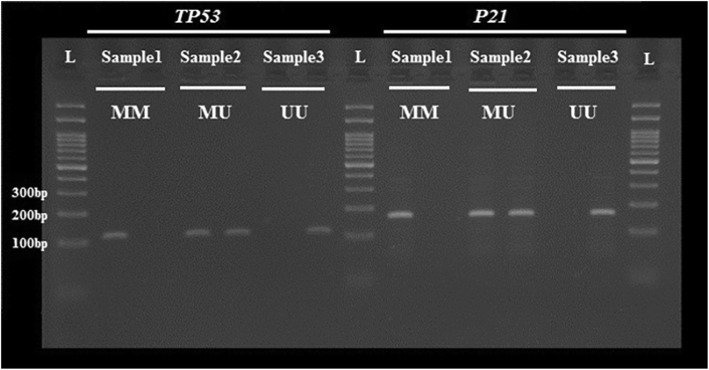
Methylation specific PCR analysis of *P21* and *TP53* promoter regions from bisulfite-treated DNA

An EpiTect Bisulfite kit (Qiagen, Germany) was used for treating the placental DNA of 109 PE and 112 control women with sodium bisulfite. The methylation-specific PCR (MSP) assay was performed to analyze the alleles (methylated and unmethylated); Table 1 presents the primers for the promoter regions of *P21* and *TP53* genes [22, 23]. For MSP amplification, a final volume of 15 μL containing 5 μL of deionized water, 7 μl of hot-start PCR master mix, 1 μL of template bisulfite DNA (~ 100 ng/μL), and 1 μL of every primer, was used. The thermocycling conditions for the PCR were as follows: 35 cycles of PCR, including 35 s of denaturation at 95 °C, 35 s of annealing at 60 °C, 35 s of extension at 72 °C, and a final 10-min extension for all primer sets at 72 °C (Fig. 1).

### Real-time PCR assay

To isolate total RNA from the placenta, we used RNX-Plus (Sinaclon, Tehran, Iran). Moreover, PrimeScript 1st strand cDNA synthesis kit (Takara Bio, Shiga, Japan) was used to generate cDNA according to the manufacturer’s instructions. An ABI PRISM 7500 RT-PCR system (Applied Biosystems) with SYBR Green was used to evaluate mRNA expression. For amplification, a reaction mixture (20 μl) of SYBR Green/high ROX (10 μl; Amplicon), 2 μl of cDNA solution, 7 μl of nuclease-free water, and 10 pmol of each primer [23–25] was used. Analysis of each sample was performed in triplicate. To determine the relative expression of mRNA in the target genes, the 2^-ΔΔCt^ method was applied, then, the expression levels were normalized to a housekeeping gene; ΔCt was the difference between *β-actin* (control) and *P21*/*TP53* genes.

### Statistical analysis

To compare the clinical parameters between the groups, Chi square was used. Using logistic regression analysis, the relationship between case–control status and genetic variants was also determined after adjustment for age, BMI and ethnicity. To determine the significant allelic and genotypic associations, the odds ratios (ORs) were measured at a 95% CI (confidence interval). The effects of polymorphism and methylation on mRNA expression were examined by the One-Way ANOVA test. Haplotype analysis was conducted using online SHEsisPlus software [26]. The Posthoc Bonferroni correction was performed to confirm the findings. To analyze data, SPSS version 20.0 was used at a significance level of 0.05.

## Results

### Population characteristics

Table 2 presents the clinical and demographic data of 454 pregnant women (228 normotensive women and 226 women with PE). The mean of maternal age and BMI were similar between two groups. The ethnicity did not differ between PE and control women. The PE group had a significantly lower gestational age and birth weight, while SBP, DBP and the frequency of primiparity were significantly higher.

**Table 2:** Demographic characteristics of PE women and controls

Variable	Controls *n* = 228	PE *n* = 226	*P*-value
Maternal age(mean ± SD, years)	28.3 ± 6.4	27.5 ± 6.2	0.2
BMI(kg/m2)	26.5 ± 3.1	27.03 ± 3.7	0.1
Gestation age(mean ± SD, days)	273 ± 18	254 ± 24	< 0.0001
Birth weight (mean ± SD, g)	3079 ± 410	2873 ± 498	< 0.0001
SBP(mean ± SD, mmHg)	103 ± 13	151 ± 19	< 0.0001
DBP(mean ± SD, mmHg)	68 ± 9.2	96 ± 12.7	< 0.0001
Primiparity, n(%)	60 (26.3)	102 (45.1)	< 0.0001
Ethnicity			
*Fars*	100 (43.9)	104 (46)	0.4
*Balooch*	128 (56.1)	122 (54)	

SD: Standard Deviation

### Maternal *P21* and *TP53* polymorphisms and PE

The genotypic and allelic frequencies of *P21* and *TP53* polymorphisms in the maternal blood are shown in Table 3. The binary logistic regression model regarding the effects of genotypes on the risk of PE showed the following results:

**Table 3:** The frequency of alleles and genotypes of maternal *P21*-rs1059234, *P21*-rs1801270 and *TP53*-rs1042522 polymorphisms in PE women and controls

	PE (*n* = 226)	Control (*n* = 228)	*P*-value	OR(95% CI)	BC *P*-value
*P21-rs1059234*					
CC, n(%)	199 (88)	199 (87)	–	1	
CT, n(%)	22 (10)	28 (12)	0.43	0.7 (0.4–1.4)	0.44
TT, n(%)	5 (2)	1 (1)	0.14	5 (0.6–43.2)	0.11
Dominant (CT + TT vs. CC)			0.8	0.9 (0.5–1.6)	0.8
Recessive (TT vs. CC + CT)			0.14	5 (0.6–44.3)	0.11
C, n(%)	420 (93)	426 (93)		1	
T, n(%)	32 (7)	30 (7)	0.8	1.1 (0.6–1.8)	–
*P21-rs1801270*					
CC, n(%)	200 (89)	177 (78)	–	1	
CA, n(%)	19 (8)	49 (21)	0.0002	0.3 (0.2–0.6)	0.0001
AA, n(%)	7 (3)	2 (1)	0.16	3.1 (0.6–15.1)	0.14
Dominant (CA + AA vs. CC)			0.002	0.4 (0.3–0.7)	0.002
Recessive (AA vs. CC + CA)			0.11	3.6 (0.7–17.6)	0.09
C, n(%)	419 (93)	403 (88)		1	
A, n(%)	33 (7)	53 (12)	0.03	0.6 (0.4–0.9)	–
*TP53-rs1042522*					
GG, n(%)	65 (29)	36 (16)	–	1	
GC, n(%)	98 (43)	101 (44)	0.012	0.5 (0.3–0.9)	0.012
CC, n(%)	63 (28)	91 (40)	0.0003	0.4 (0.2–0.6)	0.0002
Dominant (GC + CC vs. GG)			0.001	0.5 (0.3–0.7)	0.001
Recessive (CC vs. GG + GC)			0.007	0.6 (0.4–0.9)	0.007
G, n(%)	228 (51)	173 (38)		1	
C, n(%)	224 (49)	283 (62)	0.0001	0.6 (0.5–0.8)	–

BC *P*-value, Bonferroni corrected *P*-value

There was no association between PE risk and *P21*-rs1059234 polymorphism in the dominant, recessive, and allelic models. The frequency of *P21*-rs1801270 CA genotype was significantly lower in the PE group, and this genotype could protect against PE susceptibility (OR = 0.3, 95% CI: 0.2–0.6, *P* = 0.0002). Moreover, in the dominant and allelic models, *P21*-rs1801270 reduced PE risk (OR = 0.4, 95% CI: 0.3–0.7, *P* = 0.002; OR = 0.6, 95% CI: 0.4–0.9, *P* = 0.03; respectively). The frequencies of *TP53*-rs1042522 GC and CC genotypes were significantly lower in the PE group than those of the controls and were associated with a decline in PE risk (OR = 0.5, 95% CI: 0.3–0.9, *P* = 0.01; OR = 0.4, 95% CI: 0.2–0.6, *P* = 0.0003). Additionally, a significant relationship was found between *TP53*-rs1042522 and PE risk reduction in the dominant, recessive, and allelic models (OR = 0.5, 95% CI: 0.3–0.7, *P* = 0.001; OR = 0.6, 95% CI: 0.4–0.9, *P* = 0.007; OR = 0.6, 95% CI: 0.5–0.8, *P* = 0.0001).

### Placental *P21* and *TP53* polymorphisms and PE

As shown in Table 4, no association was found between the risk of PE and placental *P21*-rs1059234 polymorphism. However, the CA genotype of *P21*-rs1801270 frequency was significantly lower in the placenta of PE patients and was associated with PE risk reduction (OR = 0.4, 95% CI: 0.2–0.8, *P* = 0.013). Moreover, a significant relationship was found between *P21*-rs1801270 and PE risk reduction in the dominant model (OR = 0.5, 95%CI: 0.2–0.9, *P* = 0.034). In the recessive and allelic models, the placental *P21*-rs1801270 polymorphism had no association with PE. Based on the findings, the frequency of CC genotype in the placental *TP53*-rs1042522 polymorphism was lower in the PE group compared to controls, but the difference was marginally non-significant (*P* = 0.051). However, a significant relationship was found between *TP53*-rs1042522 and PE risk reduction in the allelic model (OR = 0.7, OR: 0.4–1, *P* = 0.03).

**Table 4:** The frequency of alleles and genotypes of placental *P21*-rs1059234, *P21*-rs1801270 and *TP53*-rs1042522 polymorphisms in PE women and controls

	PE (*n* = 109)	Control (*n* = 112)	*P*-value	OR(95% CI)	BC *P*-value
*P21-rs1059234*					
CC, n(%)	89 (81.7)	97 (86.6)	–	1	
CT, n(%)	17 (15.6)	13 (11.6)	0.37	1.4 (0.6–3.1)	0.38
TT, n(%)	3 (2.8)	2 (1.8)	0.60	1.6 (0.3–10)	0.59
Dominant (CT + TT vs. CC)			0.32	1.4 (0.7–3)	0.31
Recessive (TT vs. CC + CT)			0.63	1.6 (0.2–9.5)	0.62
C, n(%)	195 (89.5)	207 (92.4)	–	1	
T, n(%)	23 (10.5)	17 (7.6)	0.3	1.4 (0.7–2.8)	–
*P21-rs1801270*					
CC, n(%)	95 (87.2)	85 (75.9)	–	1	
CA, n(%)	11 (10.1)	26 (23.2)	0.013	0.4 (0.2–0.8)	0.010
AA, n(%)	3 (2.8)	1 (0.9)	0.40	2.7 (0.3–26.3)	0.38
Dominant (CA + AA vs. CC)			0.034	0.5 (0.2–0.9)	0.031
Recessive (AA vs. CC + CA)			0.33	3.1 (0.3–30.7)	0.30
C, n(%)	201 (92)	196 (87.5)	–	1	
A, n(%)	17 (8)	28 (12.5)	0.1	0.6 (0.3–1.1)	–
*TP53-rs1042522*					
GG, n(%)	31 (28.4)	21 (18.8)	–	1	
GC, n(%)	45 (41.3)	45 (40.2)	0.27	0.7 (0.4–1.4)	0.27
CC, n(%)	33 (30.3)	46 (41.1)	0.051	0.5 (0.2–1)	0.045
Dominant (GC + CC vs. GG)			0.091	0.6 (0.3–1.1)	0.090
Recessive (CC vs. GG + GC)			0.095	0.6 (0.4–1.1)	0.094
G, n(%)	107 (49.1)	87 (38.8)	–	1	
C, n(%)	111 (50.9)	137 (61.2)	0.03	0.7 (0.4–1)	

BC *P*-value, Bonferroni corrected *P*-value

### Haplotype analysis and linkage disequilibrium of *P21* gene

Table 5 presents four haplotypes of maternal C98A (rs1801270) and C70T (rs1059234) polymorphisms, including two alleles from each polymorphism. In both groups, C_rs1059234_ C_rs1801270_ was the most common haplotype. According to the haplotype analysis, T_rs1059234C_ C_rs1801270_ haplotype was significantly higher in PE women, which was associated with a 3.4-fold increase in PE risk (OR = 3.4, 95% CI: 1.4–8.2, *P* = 0.005). Moreover, T_rs1059234C_ A_rs1801270_ haplotype was significantly higher in healthy women, which was associated with a decreased in PE risk to 0.5-fold. (OR = 0.5, 95% CI: 0.2–1, *P* = 0.03). The linkage disequilibrium was determined for *P21* polymorphisms (D’ = 0.31, r^2^ = 0.09 in the PE group vs. D’ = 0.73, r^2^ = 0.27 in the controls).

**Table 5:** Haplotype analysis of maternal *P21*-rs1059234 and *P21*-rs1801270 polymorphisms and PE risk

Haplotype *rs1059234 rs1801270*	PE n (%)	Control n (%)	*P*-value	OR (95% CI)
*CC*	398 (88)	397 (87)	0.6	1.1 (0.7–1.6)
*TA*	11 (2)	24 (5)	0.03	0.5 (0.2–1)
*CA*	22 (5)	29 (7)	0.3	0.7 (0.4–1.3)
*TC*	21 (5)	6 (1)	0.005	3.4 (1.4–8.2)

Placental *P21*-rs1059234 and *P21*-rs1801270 haplotypes analysis showed a significant association between C_rs1059234C_A _rs1801270_ haplotype and PE risk reduction (OR = 0.4, 95%CI: 0.2–0.9, *P* = 0.03) (Table 6).

**Table 6:** Haplotype analysis of placental P21-rs1059234 and P21-rs1801270 polymorphisms and PE risk

Haplotype *rs1059234, rs1801270*	PE n (%)	Control n (%)	*P*-value	OR (95% CI)
*CC*	188 (86)	188 (84)	0.6	1.2 (0.7–2)
*TA*	10 (5)	9 (4)	0.9	1 (0.4–2.6)
*CA*	7 (3)	19 (8)	0.03	0.4 (0.2–0.9)
*TC*	13 (6)	8 (4)	0.2	1.9 (0.8–4.7)

### DNA methylation of placental *P21*/*TP53* gene

Table 7 presents the promoter DNA methylation of placental *P21* and *TP53* genes in the two groups. The higher frequency of partially methylated status (UM) in *P21* promoter region and high frequency of hyper-methylated status (MM) in *TP53* promoter region was reported in the PE group, which was associated with a higher PE risk (OR = 2.9, 95%CI = 1.1–7.2, P = 0.026; OR = 9.7, 95%CI = 1.2–79.2, *P* = 0.034; respectively). In addition, the frequency of partially and hyper-methylated status (UM + MM) in *P21* and *TP53* promoter regions was significantly higher in the PE group, which was associated with 3.4 and 3-fold increase in PE risk respectively (OR = 3.4, 95% CI = 1.4–8.3; *P* = 0.009; OR = 3, 95% CI = 1.4–6.7; *P* = 0.006; respectively).

**Table 7:** The promoter methylation status of the *P21* and *TP53* genes in PE women and control group

*Methylation status*	PE (*n* = 109)	control (*n* = 112)	OR (95% CI)	*P*-value	BC *P*-value
*P21 promoter methylation*					
UU, n (%)	89 (81.7)	105 (93.8)	1	–	
UM, n (%)	17 (15.6)	7 (6.3)	2.9 (1.1–7.2)	0.026	0.021
MM, n (%)	3 (2.8)	0 (0)	–	–	
UM + MM vs UU, n (%)			3.4 (1.4–8.3)	0.009	0.006
*TP53 promoter methylation*					
UU, n (%)	84 (77.1)	102 (91.1)	1	–	
UM, n (%)	17 (15.6)	9 (8)	2.3 (1–5.4)	0.058	0.053
MM, n (%)	8 (7.3)	1 (0.9)	9.7 (1.2–79.2)	0.034	0.010
UM + MM vs UU, n (%)			3 (1.4–6.7)	0.006	0.004

U: Unmethylated: M: Methylated; BC P-value, Bonferroni corrected P-value

### Relative mRNA expression of placental *P21* and *TP53* genes

**Fig. 2:**
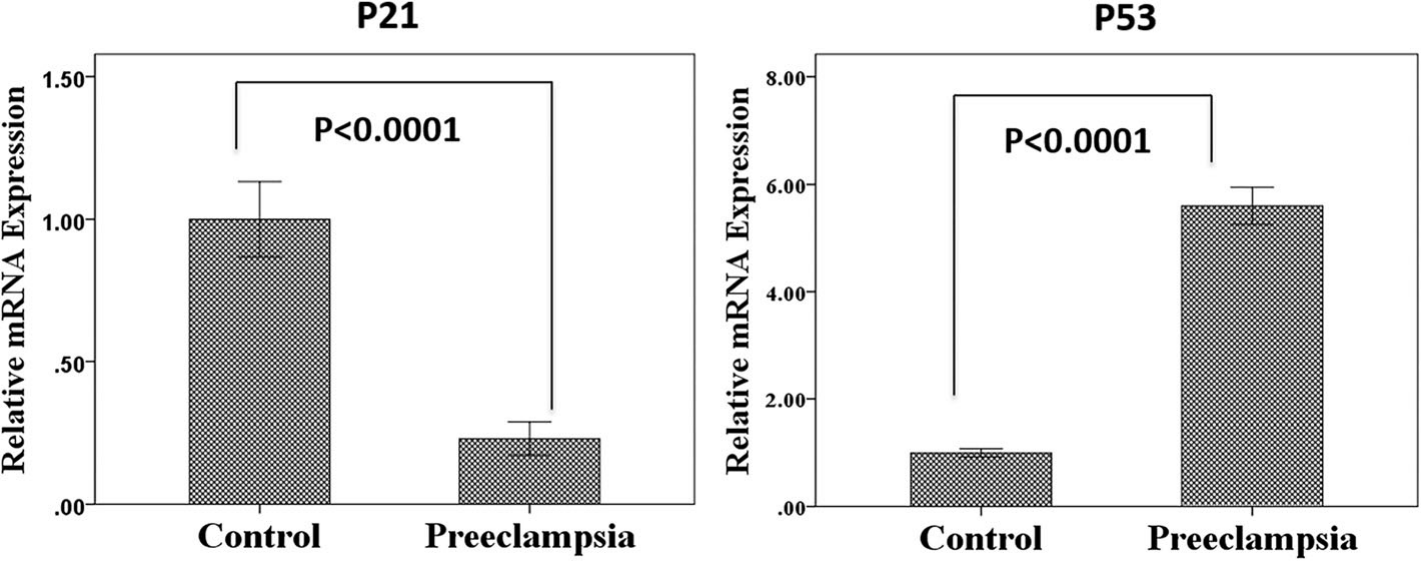
The relative mRNA expression of *P21* and *TP53* genes in PE women and controls

According to Fig. 2, the mRNA expression of the placental *P21* gene was 0.2-time in PE group compared to controls (*P* < 0.0001). Level of relative mRNA expression in the placental *TP53* gene was 5.6 times higher in the PE group compared to controls (*P* < 0.0001).

### Association between relative mRNA expression and promoter methylation of placental P21 and TP53 genes

**Fig. 3:**
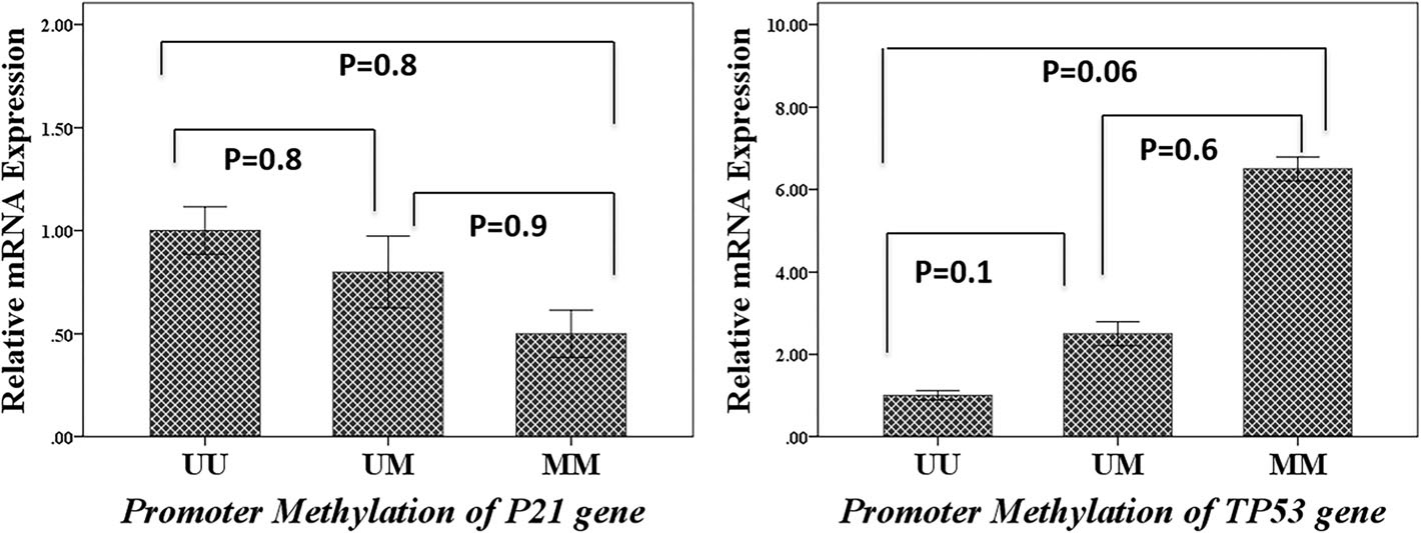
The association of relative mRNA expression and promoter methylation of placental *P21* and *TP53* genes

Although placental P21 mRNA expression was higher in un-methylated promoters in comparison with methylated promoters, the difference was not significant (*P* > 0.05). The TP53 mRNA expression was higher in the placental *TP53* methylated promoters, compared to un-methylated promoters; nonetheless, the difference was marginally not significant (*P* = 0.06) (Fig. 3).

### Association between relative mRNA expression and placental *P21* and *TP531* polymorphisms

**Fig. 4:**
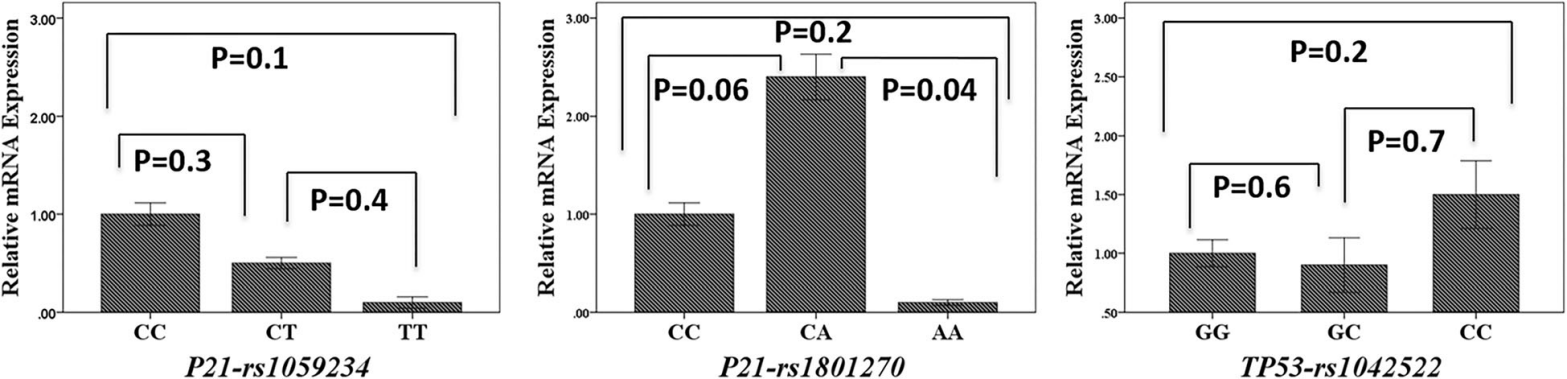
The association of relative mRNA expression and placental *P21*-rs1059234, *P21*-rs1801270 and *TP53*-rs1042522 polymorphisms

No relationship was found between *P21*-rs1059234 polymorphism and relative mRNA expression of P21. The relative mRNA expression of *P21* gene was 2.4 times higher in rs1801270 CA genotype, compared to AA genotype (*P* = 0.04). On the other hand, the relative mRNA expression of placental *TP53* gene showed no significant difference between rs1042522 genotypes (*P* > 0.05) (Fig. 4).

As shown in tables, all statistical findings remained significant after Bonferroni correction.

## Discussion

Apoptosis is the process of programmed cell death, occurring when the cells are exposed to physiological, pathogenic, or cytotoxic stimuli [27]. It also affects the life cycle of trophoblasts in the placenta [28]. Many proteins are involved in the regulation of apoptotic process, such as P53 and P21. It is well-established that the etiology of PE is related to the placenta, as the clinical symptoms are relieved after placental delivery [5]. Tomas et al. demonstrated that trophoblast apoptosis was significantly higher in PE placentas, compared with controls. Moreover, an increase in placental apoptosis and syncytial knot formation has been reported in PE pregnancies. Therefore, amplified trophoblast turnover might result in the excessive release of trophoblast into maternal circulation, producing PE symptoms [29]. P53 also contributes to the regulation of cell apoptosis [30]. According to previous studies, upregulation of P53 induces apoptosis and suppresses endothelial cell proliferation in PE pregnancies. This condition of endothelial cells in fetal and maternal circulation may contribute to the pathogenesis of PE [31]. Several mechanisms, including hypoxia and oxidative stress, can explain p53 stimulation in PE. P53 may contribute to PE at several cellular dysfunction levels,resulting in apoptosis and exaggerated autophagy [8]. Previous studies demonstrated that in complicated pregnancies, upregulation of p53 pathway leads to the buildup of downstream cell-free target genes (e.g., P21) in maternal circulation. In addition, plasma p21 mRNA expression can be indicative of complicated pregnancies [32, 33]. This case-control study compared the frequency of polymorphic variants in apoptotic genes of maternal blood and placenta of PE and normotensive pregnant women in Southeast of Iran. We found that maternal and placental *P21*-rs1801270 CA genotype was associated with a decreased PE risk (0.3 and 0.4-fold respectively). However, no association was found between maternal/placental *P21*-rs1059234 polymorphism and PE risk in the dominant, recessive, or allelic models. The maternal *TP53*-rs1042522 polymorphism was associated with the reduced risk of PE in the dominant, recessive, and allelic models. The significant association between placental *TP53*-rs1042522 and reduction in PE risk was observed in allelic model but not in dominant and recessive models. Based on the haplotype analysis, the maternal *P21* T_rs1059234_C_rs1801270_ haplotype was associated with 3.4-fold increase in PE risk, However the maternal *P21* T_rs1059234_A_rs1801270_ haplotype and placental C_rs1059234C_A _rs1801270_ haplotype led to 0.5 and 0.4-fold decrease in PE risk, respectively. Although some studies have highlighted the importance of polymorphic variants in apoptotic pathways during pregnancy, no study has yet examined the effects of maternal and placental *P21*-rs1059234 and *P21*-rs1801270 polymorphisms on the risk of PE. In addition, no study has investigated the placental *TP53*-rs1042522 polymorphism as a possible risk factor for PE. With respect to the *TP53* gene, some studies have reported a relationship between *TP53* gene SNPs and idiopathic recurrent miscarriage [34], recurrent pregnancy loss [35], uterine leiomyoma susceptibility [12], and endometriosis-associated infertility [36]. Moreover, Busatto et al. analyzed the *TP53*-rs1042522 gene polymorphism in 99 healthy pregnant and 119 PE women in Brazil. Inconsistent with our observations, the case and control groups were not significantly different [17]. The association between *P21* polymorphisms and susceptibility to uterine leiomyoma [14], late-onset Alzheimer’s disease [37], and cancer [38] has been investigated in the literature; however, the effects of *P21* polymorphisms on diseases are controversial. In addition, we found that level of relative placental mRNA expression in *TP53* gene was 5.6 times higher in the PE group (*P* < 0.0001), however, TP53 mRNA expression showed no significant difference between rs1042522 genotypes. In the placental *P21* gene, the relative mRNA expression was significantly reduced in the PE group, compared to controls (P < 0.0001). The mRNA expression of rs1801270 CA genotype was 2.4 times higher than that of AA genotype. Hyper-methylation of *P21* and *TP53* genes (UM + MM) in the promoter region was associated with a 3.4- fold and 3-fold increase in PE risk, respectively, however, no association was found between P21 and TP53 mRNA expression and promoter methylation. In this regard, Gao et al., after culturing human umbilical cord vein endothelial cells (HUVECs) from normal and PE pregnancies, evaluated cell growth. To identify the distribution of cell cycle and apoptosis, flowcytometric assessment was also performed. Based on the findings, cell growth was majorly repressed, while G1 arrest and apoptosis was increased in cultured HUVECs from PE pregnancies compared to normotensive pregnancies (controls). Since P53 protein was upregulated in HUVECs from PE pregnancies, G1 arrest, followed by P21 upregulation and downregulation of cyclin E was induced [39]. Sharp et al. found that the protein expression of p53 and p21 was increased significantly in the villous trophoblasts of PE-affected placental villi [8]. However, no significant change in the expression of apoptotic genes, such as p53, was observed in a study conducted by Mendilcioglu et al. [40]. Londero et al. reported a change in the immunohistochemical expression of cellular senescence and DNA damage markers, including *Tp53* and *p21* in the trophoblast and the placentas of PE [41]. Ashur-Fabian et al. showed the higher prevalence of cell-free plasma expressions of *hif1α* and *P21* genes in pregnancies affected by hypoxia and/or intrauterine growth restriction [33]. In addition, Davies et al. recently showed that P21 in early-onset PE placentas was significantly higher than that of controls [42]. Although the association between *P21* and *TP53* promoter methylation and PE has not been examined, several studies have confirmed the effect of *TP53* and *P21* promoter methylation on various diseases such as cancer [24, 43–45]. Indeed, some studies have confirmed the relationship between methylation of several genes and PE susceptibility [18, 46, 47].

## Conclusions

In conclusion, our results suggest that *TP53* and *P21* polymorphisms could affect the risk of PE. The decreased risk of PE was associated with *P21*-rs1801270 and *TP53*-rs1042522 polymorphisms. The risk of PE increased 3.4 times in the presence of maternal *P21* T_rs1059234_C_rs1801270_ haplotype and decreased to 0.5 and 0.4-fold in the presence of maternal T_rs1059234_A_rs1801270_ and placental C_rs1059234C_A _rs1801270_ haplotypes, respectively. *P21*-rs1801270 was associated with decreased P21 mRNA expression. In the promoter region, hyper-methylation of *P21* and *TP53* genes increased the risk of PE. However, there was no relationship between P21/TP53 mRNA expression and promoter methylation. Therefore, studies in different ethnic populations with a larger sample size should be performed to validate these findings and elucidate the molecular mechanisms of apoptosis involved in the development of PE.

## Supplementary information

**Additional file 1: Table S1.** The frequency of alleles and genotypes of maternal *P21*-rs1059234, *P21*-rs1801270 and *TP53*-rs1042522 polymorphisms in PE women and controls **Table S2.** The frequency of alleles and genotypes of placental *P21*-rs1059234, *P21*-rs1801270 and *TP53*-rs1042522 polymorphisms in PE women and controls **Table S3.** The promoter methylation status of the *P21* and *TP53* genes in PE women and control group (DOCX 18 kb)

## Data Availability

All supporting data have been shown in the manuscript.

## Abbreviations

DBP: Diastolic blood pressure
MSP: Methylation specific PCR
PCR: Polymerase chain reaction
PE: Preeclampsia
RFLP: Restriction fragment length polymorphism
SBP: systolic blood pressure
