# Supplementary material for: Analysis of polymorphisms, promoter methylation, and mRNA expression profile of maternal and placental P53 and P21 genes in preeclamptic and normotensive pregnant women
Source: J Biomed Sci. 2019 Nov 8;26:92. doi: 10.1186/s12929-019-0586-x (PMC6842146; doi:10.1186/s12929-019-0586-x)
Supplement: Supplementary file 1 — Additional file 1: Table S1. The frequency of alleles and genotypes of maternal P21-rs1059234, P21-rs1801270 and TP53-rs1042522 polymorphisms in PE women and controls Table S2. The frequency of alleles and genotypes of placental P21-rs1059234, P21-rs1801270 and TP53-rs1042522 polymorphisms in PE women and controls Table S3. The promoter methylation status of the P21 and TP53 genes in PE women and control group (DOCX 18 kb) [file 12929_2019_586_MOESM1_ESM.docx]

**Table 1:** The frequency of alleles and genotypes of maternal *P21*-rs1059234, *P21*-rs1801270 and *TP53*-rs1042522 polymorphisms in PE women and controls

|  | **PE**  **(n=23)** | **Control**  **(n=26)** | **P-value** | **OR(95% CI)** |
| --- | --- | --- | --- | --- |
| ***P21-rs1059234*** |  |  |  |  |
| CC, n(%) | 22 (95.7) | 22 (84.6) |  | 1 |
| CT, n(%) | 1 (4.3) | 4 (15.4) | 0.2 | 0.2 (0.02-2.4) |
| TT, n(%) | 0 | 0 | - | - |
| Dominant (CT+TT vs. CC) |  |  |  |  |
| Recessive (TT vs. CC+CT) |  |  | - | - |
| C, n(%) | 45 (97.8) | 48 (92.3) |  |  |
| T, n(%) | 1 (2.2) | 4 (7.7) | 0.2 | 0.3 (0.03-2.5) |
| ***P21-rs1801270*** |  |  |  |  |
| CC, n(%) | 21 (91.3) | 22 (84.6) |  | 1 |
| CA, n(%) | 1 (4.3) | 4 (15.4) | 0.2 | 0.3 (0.03-2.5) |
| AA, n(%) | 1 (4.3) | 0 | 0.5 | 3.1 (0.1-81.4) |
| Dominant (CA+AA vs. CC) |  |  | 0.5 | 0.5 (0.09-3.2) |
| Recessive (AA vs. CC+CA) |  |  | - | - |
| C, n(%) | 43 (93.5) | 48 (92.3) |  |  |
| A, n(%) | 3 (6.5) | 4 (7.7) | 0.8 | 0.8 (0.2-3.9) |
| ***TP53-rs1042522*** |  |  |  |  |
| GG, n(%) | 9 (39.1) | 7 (26.9) |  | 1 |
| GC, n(%) | 7 (30.4) | 5 (19.2) | 0.9 | 1.1 (0.2-5) |
| CC, n(%) | 7 (30.4) | 14 (53.8) | 0.2 | 0.4 (0.1-1.5) |
| Dominant (GC+CC vs. GG) |  |  | 0.4 | 0.6 (0.2-2) |
| Recessive (CC vs. GG+GC) |  |  | 0.1 | 0.4 (0.1-1.2) |
| G, n(%) | 25 (54.3) | 19 (36.5) |  |  |
| C, n(%) | 21 (45.7) | 33 (63.5) | 0.08 | 0.5 (0.2-1.1) |

**Table 2:** The frequency of alleles and genotypes of placental *P21*-rs1059234, *P21*-rs1801270 and *TP53*-rs1042522 polymorphisms in PE women and controls

|  | **PE**  **(n=23)** | **Control**  **(n=28)** | **P-value** | **OR(95% CI)** |
| --- | --- | --- | --- | --- |
| ***P21-rs1059234*** |  |  |  |  |
| CC, n(%) | 17 (73.9) | 23 (82.1) |  | 1 |
| CT, n(%) | 6 (26.1) | 5 (17.9) | 0.5 | 1.6 (0.4-6.2) |
| TT, n(%) | 0 | 0 |  |  |
| Dominant (CT+TT vs. CC) |  |  |  |  |
| Recessive (TT vs. CC+CT) |  |  | - | - |
| C, n(%) | 40 (87) | 51 (91.1) |  | 1 |
| T, n(%) | 6 (13) | 5 (8.9) | 0.5 | 1.5 (0.4-5.4) |
| ***P21-rs1801270*** |  |  |  |  |
| CC, n(%) | 20 (87) | 21 (75) |  | 1 |
| CA, n(%) | 3 (13) | 7 (25) | 0.3 | 0.4 (0.1-2) |
| AA, n(%) | 0 | 0 |  |  |
| Dominant (CA+AA vs. CC) |  |  |  |  |
| Recessive (AA vs. CC+CA) |  |  | - | - |
| C, n(%) | 43 (93.5) | 49 (87.5) |  | 1 |
| A, n(%) | 3 (6.5) | 7 (12.5) | 0.3 | 0.5 (0.1-2) |
| ***TP53-rs1042522*** |  |  |  |  |
| GG, n(%) | 8 (34.8) | 7 (25) |  | 1 |
| GC, n(%) | 7 (30.4) | 7 (25) | 0.9 | 09 (0.2-3.8) |
| CC, n(%) | 8 (34.8) | 14 (50) | 0.3 | 0.5 (0.1-1.9) |
| Dominant (GC+CC vs. GG) |  |  | 0.4 | 0.6 (0.2-2.1) |
| Recessive (CC vs. GG+GC) |  |  | 0.3 | 0.5 (0.2-1.6) |
| G, n(%) | 23 (50) | 21 (37.5) |  | 1 |
| C, n(%) | 23 (50) | 35 (62.5) | 0.2 | 0.6 (0.3-1.3) |

**Table 3:** The promoter methylation status of the *P21* and *TP53* genes in PE women and control group

| **P-value** | **OR (95% CI)** | **control**  **(n=28)** | **PE**  **(n=23)** | ***Methylation status*** |
| --- | --- | --- | --- | --- |
| **P21 promoter methylation** | | | | |
| 1 |  | 26 (92.9) | 17 (73.9) | UU, n (%) |
| 0.08 | 4.6 (0.8-25.4) | 2 (7.1) | 6 (26.1) | UM, n (%) |
|  | - | 0 | 0 | MM, n (%) |
| 0.08 | 4.6 (0.8-25.4) |  |  | UM+MM vs UU, n (%) |
| **TP53 promoter methylation** | | | | |
|  |  | 25 (89.3) | 15 (65.2) | UU, n (%) |
| 0.07 | 3.9 (0.9-17.3) | 3 (10.7) | 7 (30.4) | UM, n (%) |
| 0.3 | 4.9 (0.2-128.8) | 0 | 1 (4.3) | MM, n (%) |
| 0.05 | 4.4 (1-19.4) |  |  | UM+MM vs UU, n (%) |

U: Unmethylated, M: Methylated
